# Local Yield Stress Distribution of HDPE Pipe Butt Fusion Joints Measured by a Small Punch Test

**DOI:** 10.3390/polym18172094

**Published:** 2026-08-28

**Authors:** Chunmei Bai, Xiehang Duan, Zhuang Hu, Huan Sheng Lai, Yuntao Zhong, Peinan Du

**Affiliations:** 1 Architecture and Civil Engineering Institute, Guangdong University of Petrochemical Technology, Maoming 525000, China; 2 Sinochem Quanzhou Petrochemical Co., Ltd., Quanzhou 362103, China; 3 Sino-French Institute of Nuclear Engineering and Technology, Sun Yat-sen University, Zhuhai 519082, China; 4 Science and Technology on Reactor Fuel and Materials Laboratory, Nuclear Power Institute of China, Chengdu 610213, China

**Keywords:** HDPE, welded joint, yield stress, butt fusion welding, small punch test

## Abstract

Butt fusion welding is the primary connection technology for high-density polyethylene (HDPE) pipelines, yet welded joints are the most vulnerable location for pipeline service failures. Conventional uniaxial tensile tests can only obtain the average strength of the whole weldment and fail to capture the spatial variation of local yield stress across narrow joint zones. In this study, small punch tests (SPTs) combined with finite element parametric simulations were adopted to quantitatively characterize the position-dependent yield stress of HDPE pipe butt fusion joints. Simulations were performed to establish a correlation formula between SPT peak load–displacement parameters and HDPE yield stress, and the effects of material properties and contact friction on empirical coefficients were systematically analyzed. The calibrated formula under a friction coefficient of 0.5 was verified against uniaxial tensile data of base HDPE, showing acceptable prediction accuracy. For joints fabricated following a ISO 21307 standard, the yield stress of the fusion zone was higher than that of the base pipe material. The maximum yield stress appeared at 3 mm offset from the weld center plane, approximately 4% higher than the base matrix. This study validated the feasibility of using SPT to realize micro-sampling localized mechanical measurement of HDPE welded joints, offering a convenient testing method for on-site quality assessment of polyethylene pipeline welds.

## 1. Introduction

High-density polyethylene (HDPE) pipes have become the dominant structural medium for urban water supply and natural gas transmission systems, owing to their outstanding corrosion resistance, low installation cost, high toughness and long service life [1,2]. Butt fusion welding is the most widely adopted connection technique for HDPE pipelines, which realizes molecular diffusion and interfacial bonding by heating and pressurizing pipe ends. However, numerous engineering accidents and laboratory characterizations have confirmed that welded joints act as the weakest link of the whole pipeline structure, where most creep rupture, slow crack growth and tensile failure incidents initiate [3,4]. Therefore, accurate evaluation of the mechanical properties at different positions of HDPE pipe butt fusion welded joints is essential to assess welding quality and long-term pipeline service safety.

Tensile properties are the basic mechanical properties. Uniaxial tensile testing is the conventional method to measure the tensile properties of HDPE pipe butt fusion welded joints. In standard tensile tests, a complete welded joint is placed at the center of dumbbell specimens to obtain the overall bearing capacity of the weldment [5,6,7,8]. However, this test method solely assesses the entire quality of the welded joints and does not provide precise measurement of their tensile properties at different locations of welded joints. Given the small volume of the welded joints, a test method with miniature specimen should be employed to examine their tensile properties.

The small punch test (SPT) has been extensively developed for localized mechanical characterization of metallic materials over the past two decades. Standard SPT specimens are circular thin disks with a diameter of 8 mm and thickness of 0.5 mm [9], requiring only an extremely small sampling area from target components. Existing studies have verified the capability of SPT to quantify tensile properties [10], fracture toughness [11,12], and creep properties [13,14,15,16] of various metal alloys. A combined SPT and stepped isostress method has been developed to investigate the creep behavior of HDPE pipe [17]. In addition, for metallic materials, independent empirical correlation formulas are established to calculate yield strength and ultimate tensile strength from SPT load–displacement curves, and the corresponding fitting coefficients are mainly dependent on fixture geometry and material ductility [18,19,20,21,22]. Therefore, SPT is theoretically employed to evaluate the tensile properties of polyethylene materials.

However, the constitutive deformation characteristics of polymers differ fundamentally from metals, which prevents the direct transplantation of metal-derived SPT calibration models to polyethylene materials. Firstly, metals feature distinct yield strength and ultimate tensile strength, while HDPE only presents a single yield point corresponding to the peak stress on engineering tensile curves. Secondly, prior polymer SPT research on PLA and PET found two separate peak loads on load–displacement curves, and the first peak load was recommended for yield stress calculation [23]; by contrast, the load response rule of HDPE under SPT biaxial loading remains unclear. In addition, polymer ductility is far higher than that of metals, which inevitably changes the contact friction state and deformation evolution during SPT loading, further invalidating empirical coefficients fitted from metal specimens. At present, few investigations have established a targeted SPT quantitative calculation method for the yield stress of HDPE materials, and there is a lack of research adopting SPT to map the local yield stress distribution across HDPE pipe butt fusion welded joints.

To fill the above research gaps, this work proposed a combined numerical-experimental framework based on SPT to quantitatively characterize the position-dependent yield stress of HDPE pipe butt fusion welded joints. Finite element parametric simulations were first performed to establish a dedicated correlation equation between SPT characteristic load-displacement parameters and HDPE yield stress, and the effects of material mechanical parameters and contact friction on empirical fitting coefficients were systematically analyzed. Subsequently, SPT experiments were carried out on specimens extracted from the weld center, heat-affected zone and base material of HDPE pipe butt fusion welded joints to acquire the spatial distribution of yield stress. The findings of this study could provide a miniature-sampling testing approach for localized mechanical performance evaluation of HDPE pipe butt fusion welded joints and offer reference for the quality inspection of polyethylene pipeline engineering.

## 2. Determination of Yield Stress via Small Punch Test

**Figure 1:**
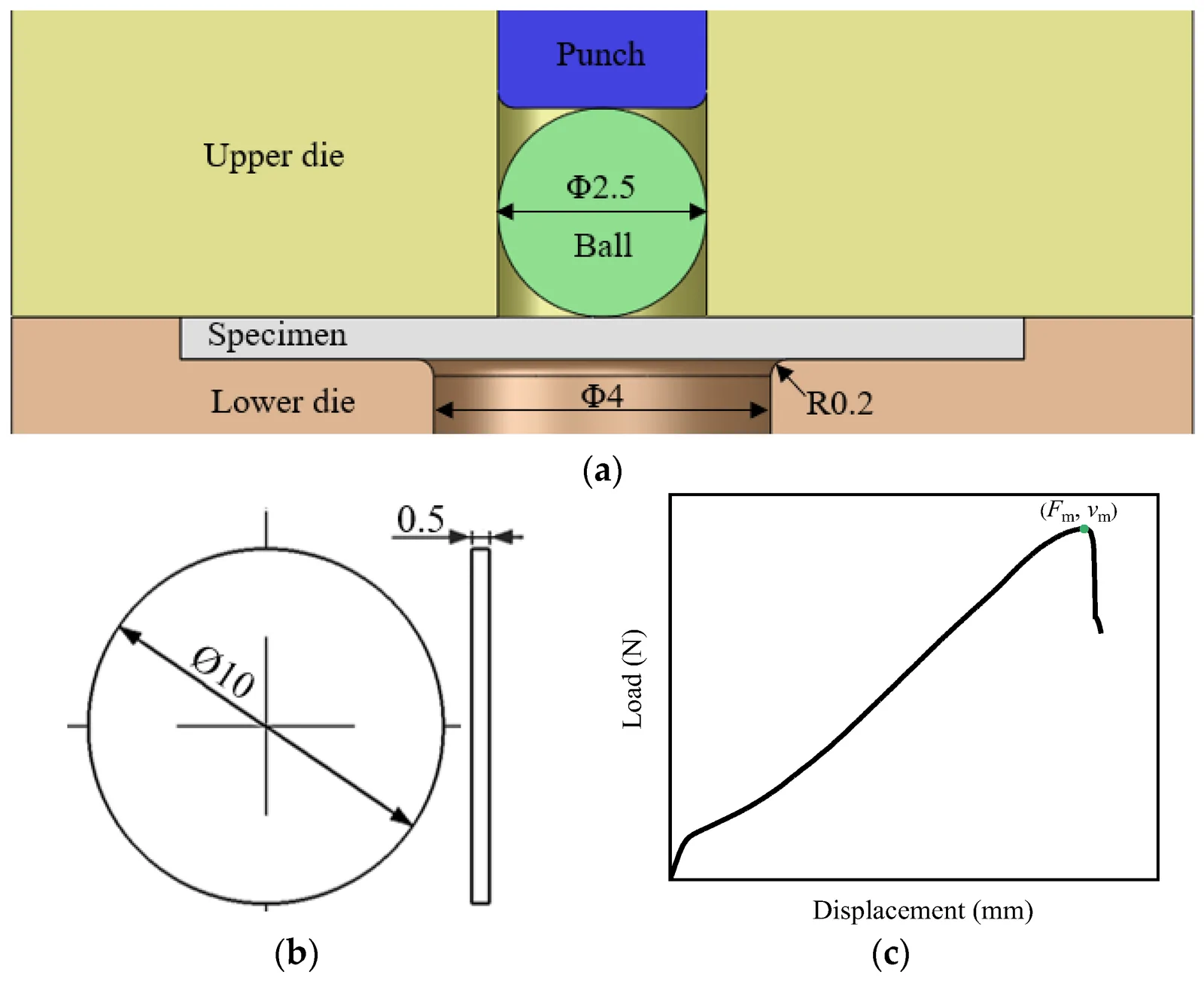
SPT set-up, specimen dimensions, and load-displacement curve: (**a**) dimensions of the SPT set-up, (**b**) dimensions of the specimen, (**c**) typical experimental load-displacement curve.

The geometric dimensions of the standard SPT set-up and specimen are shown in Figure 1a and Figure 1b, respectively [9]. During testing, the thin disk specimen is rigidly clamped between upper and lower dies, and the rigid spherical indenter is in contact with both the cylindrical punch and the specimen. A compressive load is applied to the specimen via the cylindrical punch, which imposes displacement-controlled loading at a constant rate. A representative load–displacement curve recorded from SPT is shown in Figure 1c.

For HDPE, the yield stress $\sigma_{y}$ corresponds to the peak stress on engineering tensile curves, analogous to the ultimate tensile strength of metallic alloys. Accordingly, the correlation formula originally developed to calculate metal ultimate tensile strength is adopted to compute HDPE yield stress [24]:

(1) $$\sigma_{y}=\beta_{\mathrm{Rm}}\frac{F_{m}}{h_{0}v_{m}}+\beta_{0}$$

where $h_{0}$ is the initial specimen thickness, $F_{m}$ is the maximum load during the test, $v_{m}$ is the displacement at $F_{m}$, and $\beta_{0}$ and $\beta_{\mathrm{Rm}}$ are empirical factors dependent on the set-up and material properties. The values of $\beta_{0}$ and $\beta_{\mathrm{Rm}}$ can be determined using experiments or finite element parametric simulations.

## 3. Finite Element Simulation

### 3.1. Finite Element Model

**Figure 2:**
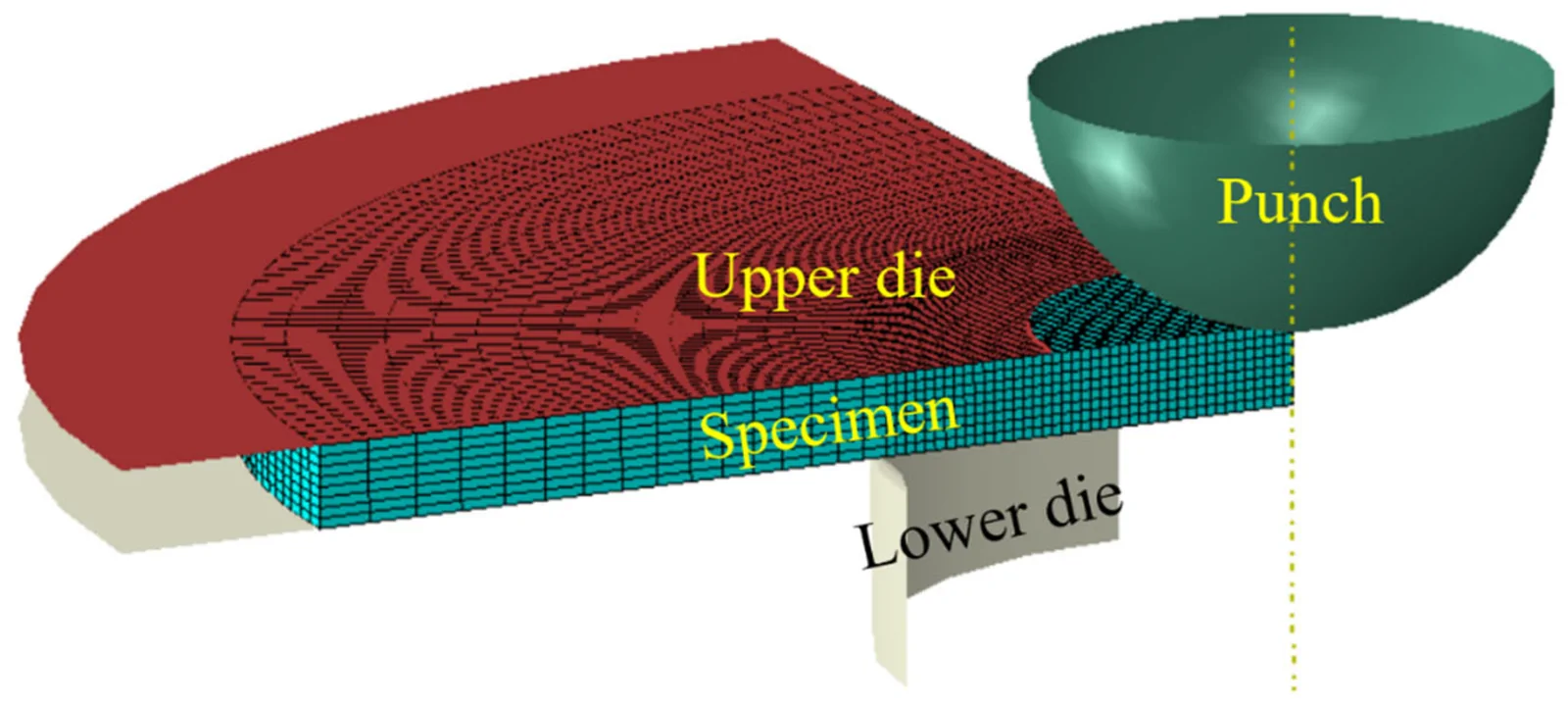
Quarter-symmetric finite element model of the SPT assembly.

Abaqus was used to conduct parametric SPT simulations for determining the values of $\beta_{0}$ and $\beta_{\mathrm{Rm}}$. Geometric symmetry allows only a quarter of the full specimen-fixture assembly to be modeled, as shown in Figure 2. The mesh within the punch contact region was refined to 0.05 mm × 0.05 mm × 0.05 mm using the linear brick elements C3D8R. The spherical punch, upper die and lower die were defined as rigid bodies. To investigate the friction effect on the values of $\beta_{\mathrm{Rm}}$ and $\beta_{0}$, uniform friction coefficients of μ = 0.3, 0.4, and 0.5 were assigned to all contact interfaces between the specimen and fixture components.

**Figure 3:**
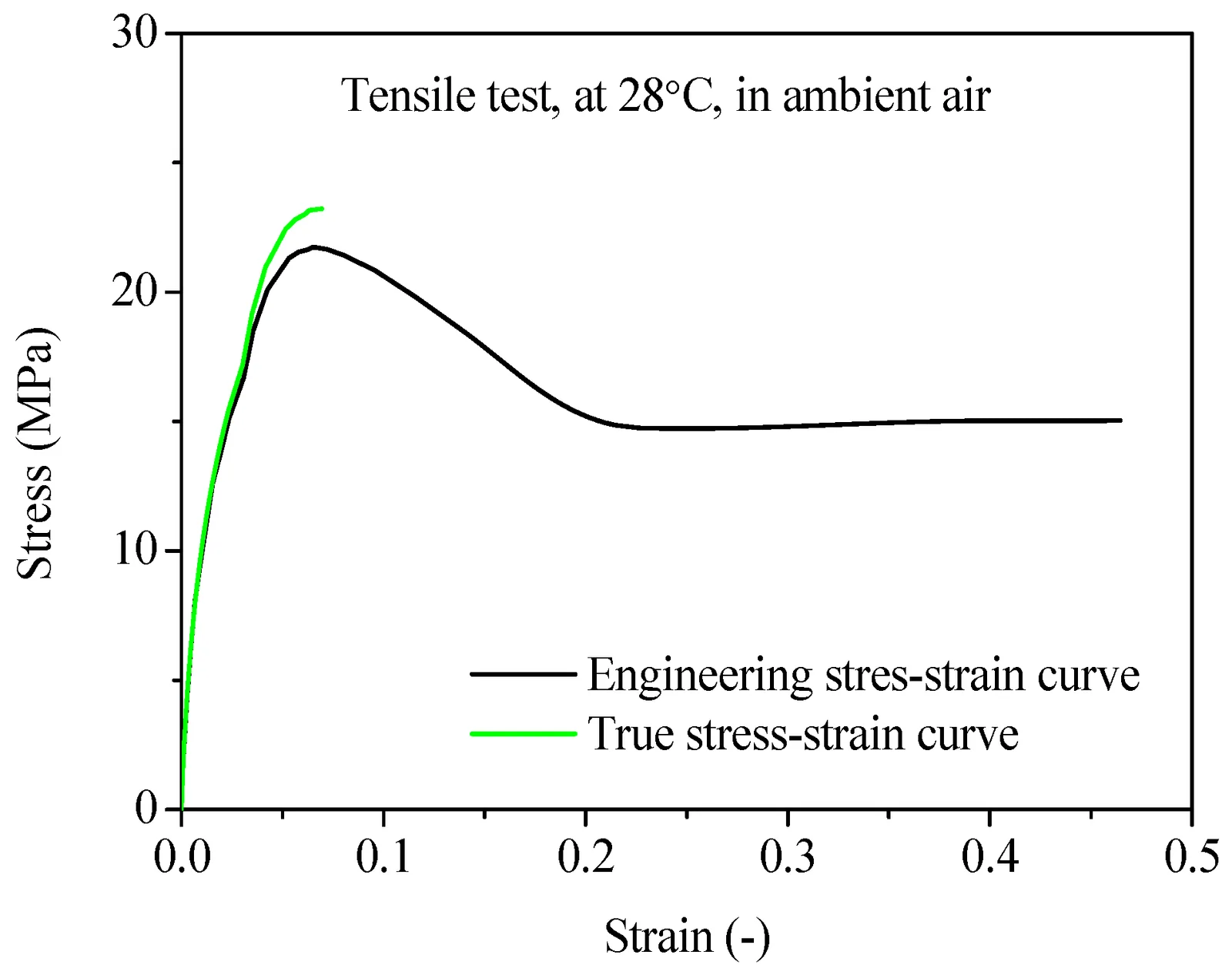
Tensile test results of HDPE pipes at 28 °C in ambient air [25].

**Figure 4:**
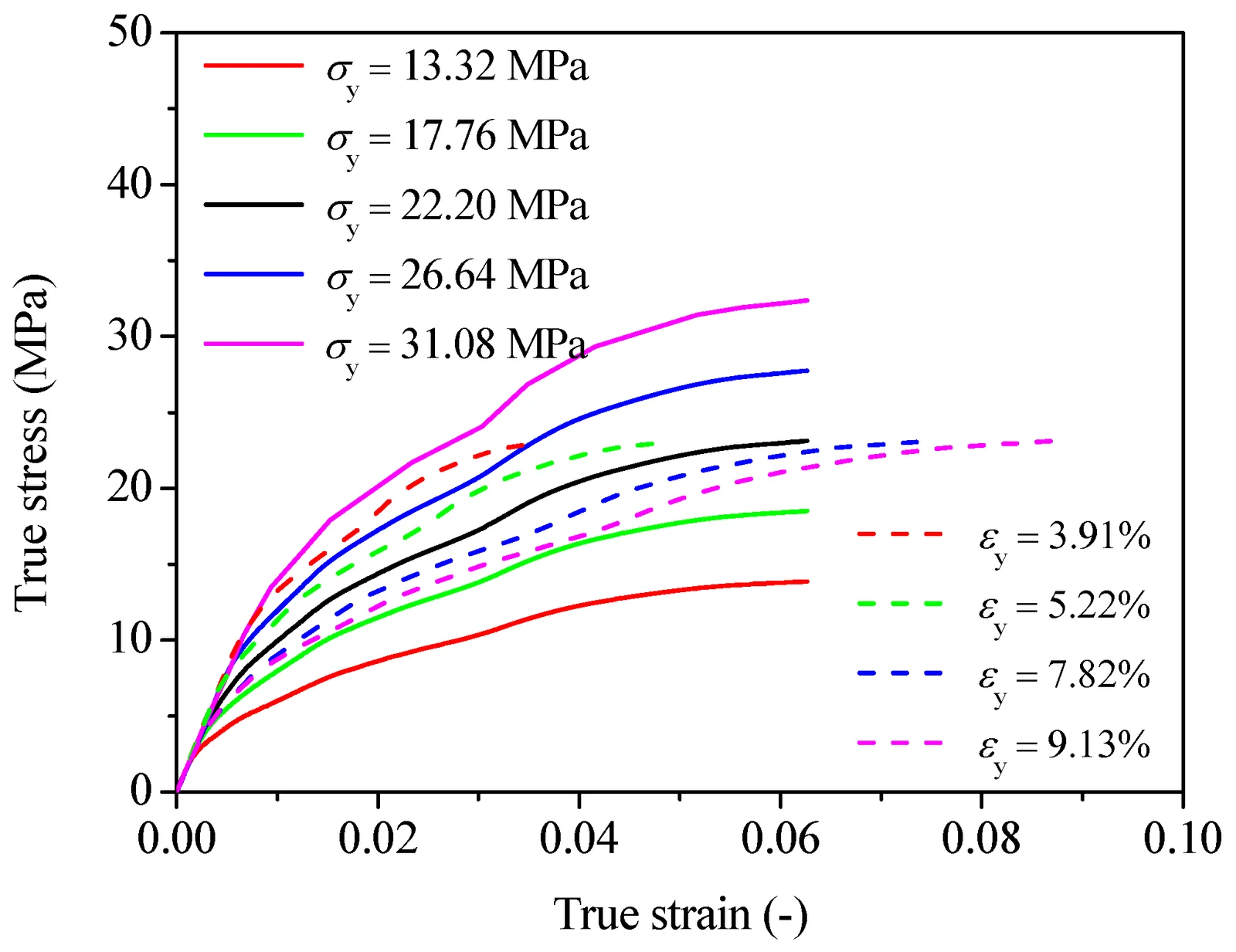
True stress–strain curves used in SPT simulations for different cases.

The uniaxial tensile response of the employed HDPE pipe at 28 °C under ambient air was investigated in our prior work (Figure 3) [25], with key material parameters: $\sigma_{y}$ = 22.2 MPa, strain corresponding to the yielding stress $\varepsilon_{y}$ = 6.52%, Poisson’s ratio *v* = 0.4, and elastic modulus *E* = 1314 MPa [25]. The experimental true stress–strain curve was taken as the baseline to generate a series of modified constitutive curves with varying values of $\sigma_{y}$ and $\varepsilon_{y}$, as shown in Figure 4. Ideal plastic behaviors were assumed once the true stress reached to the maximum value for all cases. All cases and the corresponding material inputs are listed in Table 1. The cases were divided into two groups. Group 1 (Cases 1–5): Fixed $\varepsilon_{y}$, *v*, and *E*, with variable $\sigma_{y}$–to used isolate and investigate the influence of $\sigma_{y}$ on $\beta_{0}$ and $\beta_{\mathrm{Rm}}$. Group 2 (Case 3 and Cases 6–9): Fixed $\sigma_{y}$, *v*, and *E*, with variable $\varepsilon_{y}$–to used isolate and investigate the influence of $\varepsilon_{y}$ on $\beta_{0}$ and $\beta_{\mathrm{Rm}}$. Elastic modulus and Poisson’s ratio exhibit negligible variation among commercial HDPE pipes, so their minor secondary effects on correlation coefficients were not further analyzed here.

### 3.2. Simulation Results

**Figure 5:**
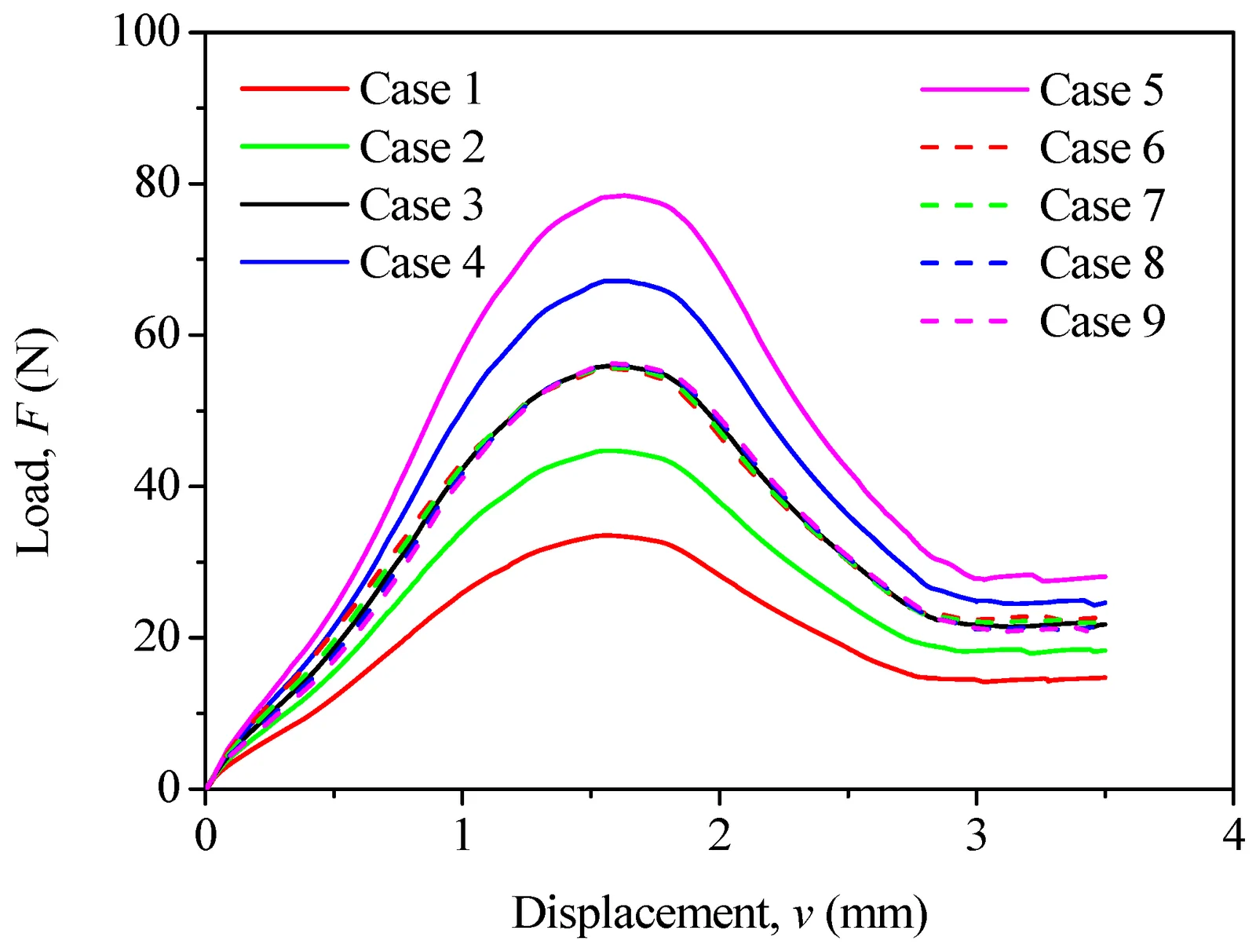
Simulated load–displacement curves for all cases.

Simulated load–displacement curves for all cases with μ = 0.3 are shown in Figure 5. Curves obtained at μ = 0.4 and 0.5 follow identical overall trends. For every case, load increased monotonically to a distinct peak $F_{m}$ before gradually decreasing to a steady plateau as displacement increased. No fracture or damage initiation criteria were incorporated into the finite element framework. Therefore, all simulations terminated when the punch displacement reached 3.5 mm.

The values of $F_{m}$ and $v_{m}$ are listed in Table 2 for all cases under three friction coefficients. Generally, the values of $F_{m}$ were less than 100 N, significantly less than typical metal materials (~1800 N) [23], which accounted for the much lower yield stress of HDPE (~25 MPa vs. ~300 MPa for metal materials). In addition, all cases demonstrated nearly identical values of $v_{m}$. The values of $v_{m}$ were slightly larger than 1.5 mm, reflecting the exceptional ductility of HDPE.

For Group 1 (Cases 1–5, fixed $\varepsilon_{y}$, *v*, and *E*), both $F_{m}$ and $v_{m}$ increased with the increase in $\sigma_{y}$ at μ = 0.3, as listed in Table 2. For Group 2 (Case 3 and Cases 6–9, fixed $\sigma_{y}$, *v*, and *E*), $F_{m}$ increased slightly with increase of $\varepsilon_{\mathrm{UTS}}$ at μ = 0.3, but the effect of $\varepsilon_{\mathrm{UTS}}$ on $v_{m}$ was almost imperceptible, as listed in Table 2. For the two groups, $F_{m}$ increased with an increase in μ, but the effect of μ on $v_{m}$ was irregular and not obvious. Therefore, $F_{m}$ was mainly affected by the values of $\sigma_{y}$ and μ, while $v_{m}$ was slightly affected by $\sigma_{y}$.

**Figure 6:**
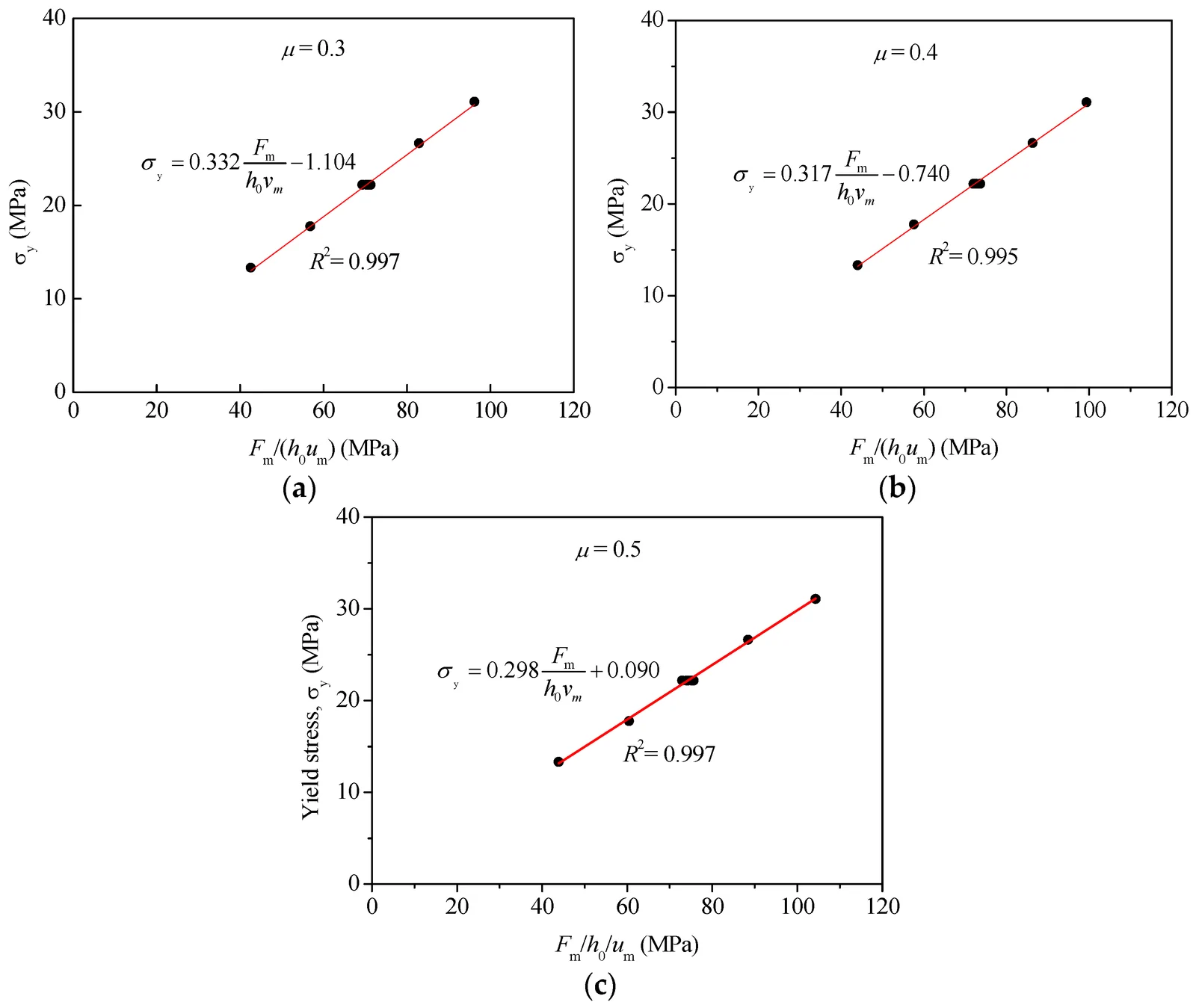
Fitted curves between the values of $\sigma_{y}$ and $F_{m}/(h_{0}u_{m})$: (**a**) μ = 0.3, (**b**) μ = 0.4, (**c**) μ = 0.5.

The values of $F_{m}/(h_{0}v_{m})$ could be calculated based on the simulation results (Table 2). Linear fitting was performed between $\sigma_{y}$ and $F_{m}/(h_{0}v_{m})$ for each value of μ, as shown in Figure 6, where *R*^2^ is the goodness of fit. All fitting coefficients of determination were *R*^2^ > 0.99. Therefore, Equation (1) provided a satisfactory fit to the data. The calibrated correlation formulas under three friction levels are listed below:

(2) $$\sigma_{y}=0.332\frac{F_{m}}{h_{0}v_{m}}-1.104\;\;\;\;\;\;\;\;\;\;\;\;\;\;\;\mathrm{for}\;\mu =0.3$$

(3) $$\sigma_{y}=0.317\frac{F_{m}}{h_{0}v_{m}}-0.740\;\;\;\;\;\;\;\;\;\;\;\;\;\;\;\mathrm{for}\;\mu =0.4$$

(4) $$\sigma_{y}=0.298\frac{F_{m}}{h_{0}v_{m}}+0.090\;\;\;\;\;\;\;\;\;\;\;\;\;\;\;\mathrm{for}\;\mu =0.5$$

## 4. A Small Punch Test of HDPE Pipe Butt Fusion Welded Joints

### 4.1. Experimental Setup and Specimen Preparation

**Figure 7:**
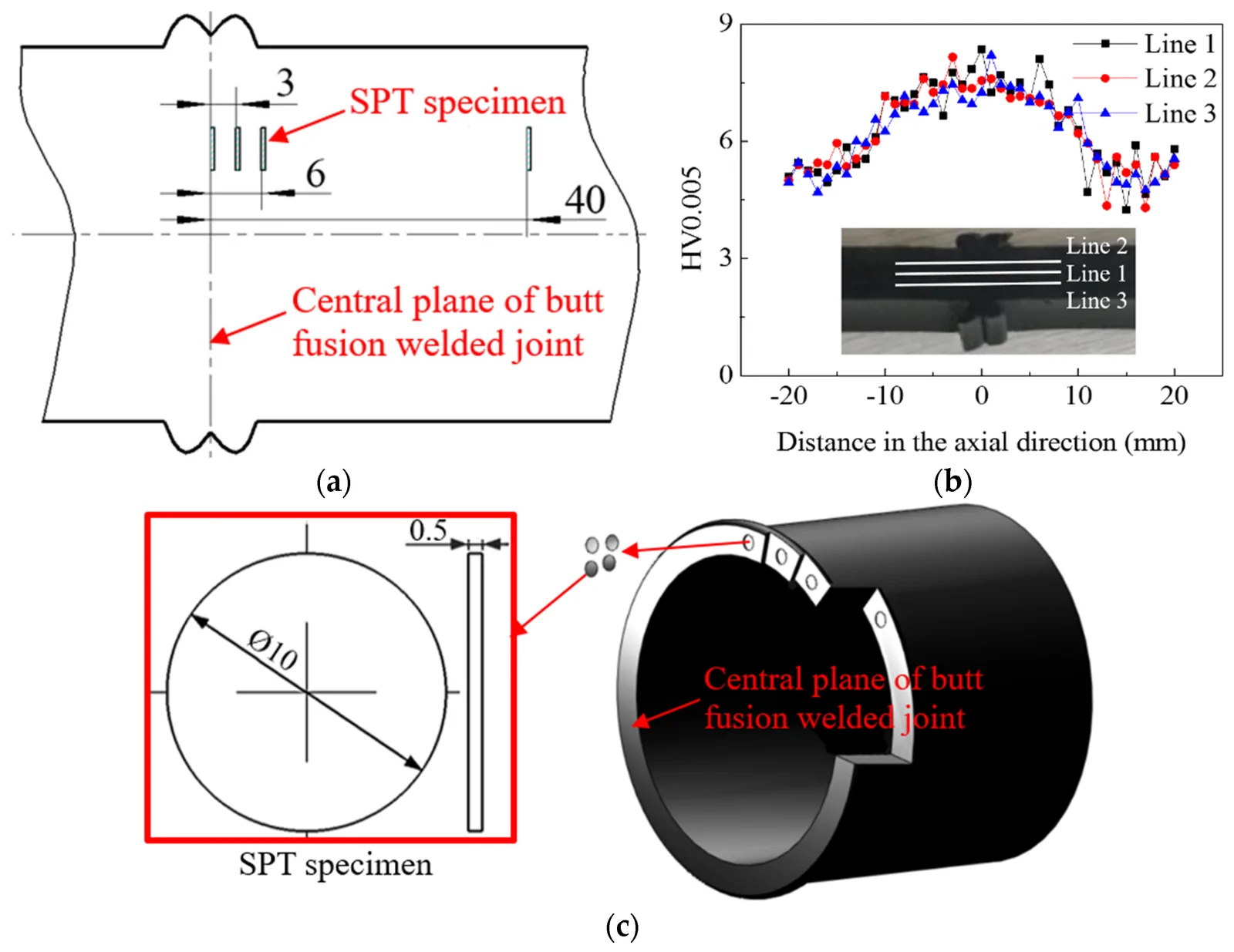
SPT specimen sampling layout: (**a**) cross-sectional positions of SPT specimens across the butt fusion welded joint; (**b**) hardness distribution along the axial direction of the HDPE pipe butt fusion welded joint [28]; (**c**) extraction of SPT specimens from the HDPE pipe butt fusion welded joint.

**Figure 8:**
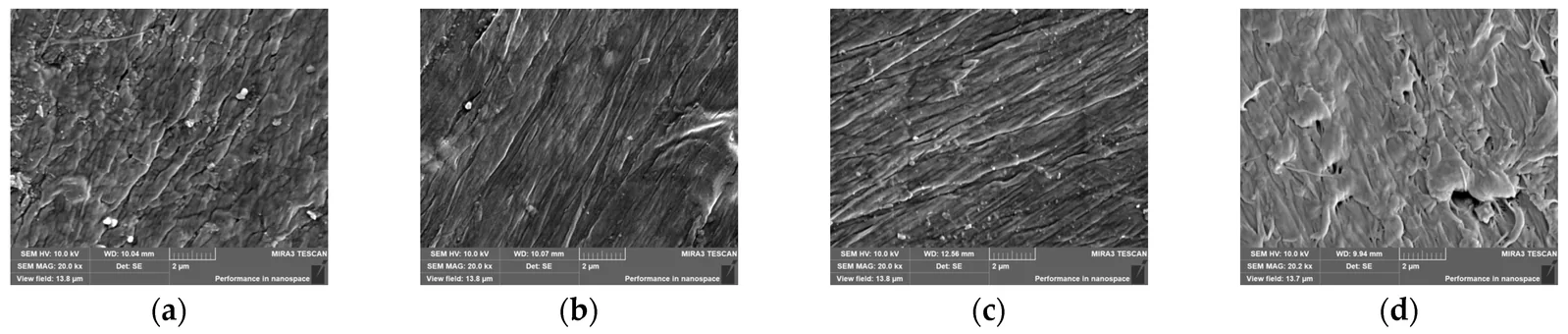
SEM micrographs of HDPE pipe butt-fusion welded joint, acquired prior to testing at axial distances of 0 mm (**a**), 3 mm (**b**), 6 mm (**c**), and 23 mm (**d**) from the weld center [27].

The HDPE pipe employed has a standard dimension ratio (SDR) of 11, with an outer diameter of 200 mm and a nominal wall thickness of 18.18 mm. Butt fusion welding was conducted strictly following ISO 21307 [26], using a butt fusion welding machine; the key welding parameters are listed in Table 3 [27]. SPT specimens were machined at four distinct radial offsets from the weld center plane, as shown in Figure 7, with all specimens extracted at the mid-wall thickness position. These locations are denoted as 0#, 3#, 6#, and 40# to represent distances of 0 mm, 3 mm, 6 mm, and 40 mm, respectively, away from the central plane of the welded joint. The hardness distribution along the axial direction of the HDPE pipe butt fusion welded joint was investigated prior to testing in our previously work [28], as shown in Figure 7b. It was observed that the hardness became constant at positions approximately 20 mm from the weld central plane. Therefore, specimen 40#—located sufficiently far from the heat-affected zone—exhibited mechanical properties representative of the base material, particularly its yield stress. In addition, the microstructure along the axial direction of the HDPE pipe butt fusion welded joint was characterized prior to testing in our previous work, as shown in Figure 8 [27]. Significant microstructural variations were observed along the axial direction of the welded joint, implying location-dependent mechanical properties within the joint.

The initial thickness of the specimens was 0.7 mm, which was subsequently wet-polished with 1200-grit silicon carbide sandpaper to achieve a uniform finished thickness of 0.5 ± 0.005 mm and a nominal diameter of 10.0 mm, as shown in Figure 7b. The dimensions of the SPT set-up are shown in Figure 1a. Displacement-controlled loading was applied at a constant crosshead speed of 1.0 mm/min. All tests were carried out inside a temperature-controlled environmental chamber at 29 ± 1 °C. Specimens were thermally stabilized for 0.5 h prior to loading to ensure uniform internal temperature distribution.

### 4.2. Small Punch Test Results

**Figure 9:**
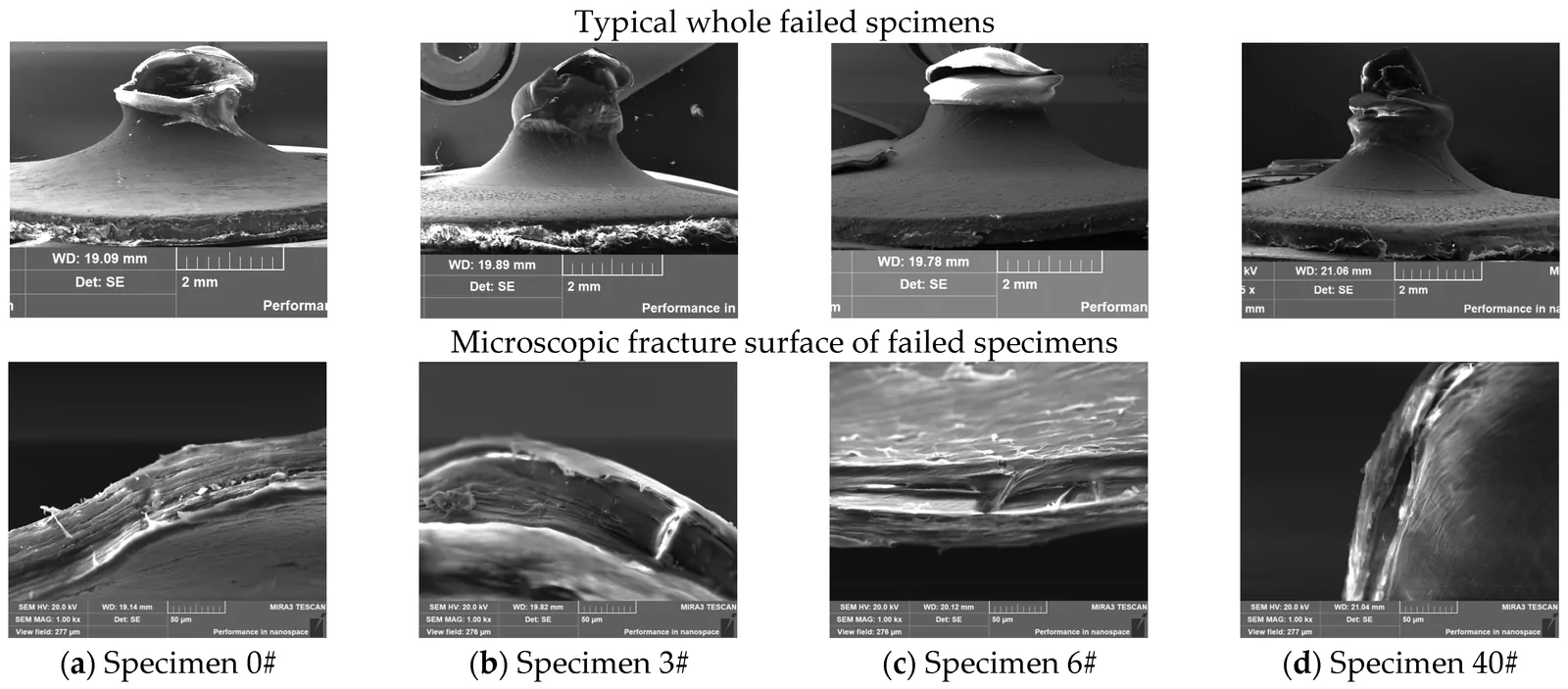
Typical failed SPT specimens extracted from distinct axial positions across the HDPE pipe butt fusion welded joint.

The overall macroscopic morphologies and corresponding high-magnification side-view fracture microstructures of the failed SPT specimens are shown in Figure 9. All specimens exhibited pronounced ductile bulging, with final rupture localized at the top dome region—consistent with the inherent high plasticity and strain-hardening capacity of HDPE. Specimen 0# displayed marked dome bulging accompanied by macroscopic tearing and extensive sheet-like delamination at the apex; high-magnification analysis revealed multi-layered interfacial separation and laminated tearing adjacent to the fracture surface. Specimen 3# showed comparable bulging height to 0#, but its apex featured irregular, non-uniform tearing; the side-view fracture morphology exhibited distinct layered cracking and shear-induced laminated deformation. Specimen 6# underwent substantial plastic bulging, with crack initiation occurring precisely at the dome apex and exhibiting relatively flat, blunt torn edges; its side-view microstructure was characterized by pronounced interlayer slip and highly stretched fibrous deformation. Specimen 40#, located farthest from the weld center, exhibited the most severe dome deformation and developed a steep, open crack at the apex; its fracture surface was dominated by shear-driven cracking, with markedly reduced laminated delamination relative to the other three specimens. Collectively, a systematic transition in failure behavior is observed with increasing distance from the weld center: specimens 0#, 3#, and 6# display progressive laminated delamination and multi-layer tearing—indicative of interfacial weakness and layer-wise decohesion—whereas specimen 40# exhibits predominantly shear-dominated fracture, reflecting robust through-thickness cohesion strength.

**Figure 10:**
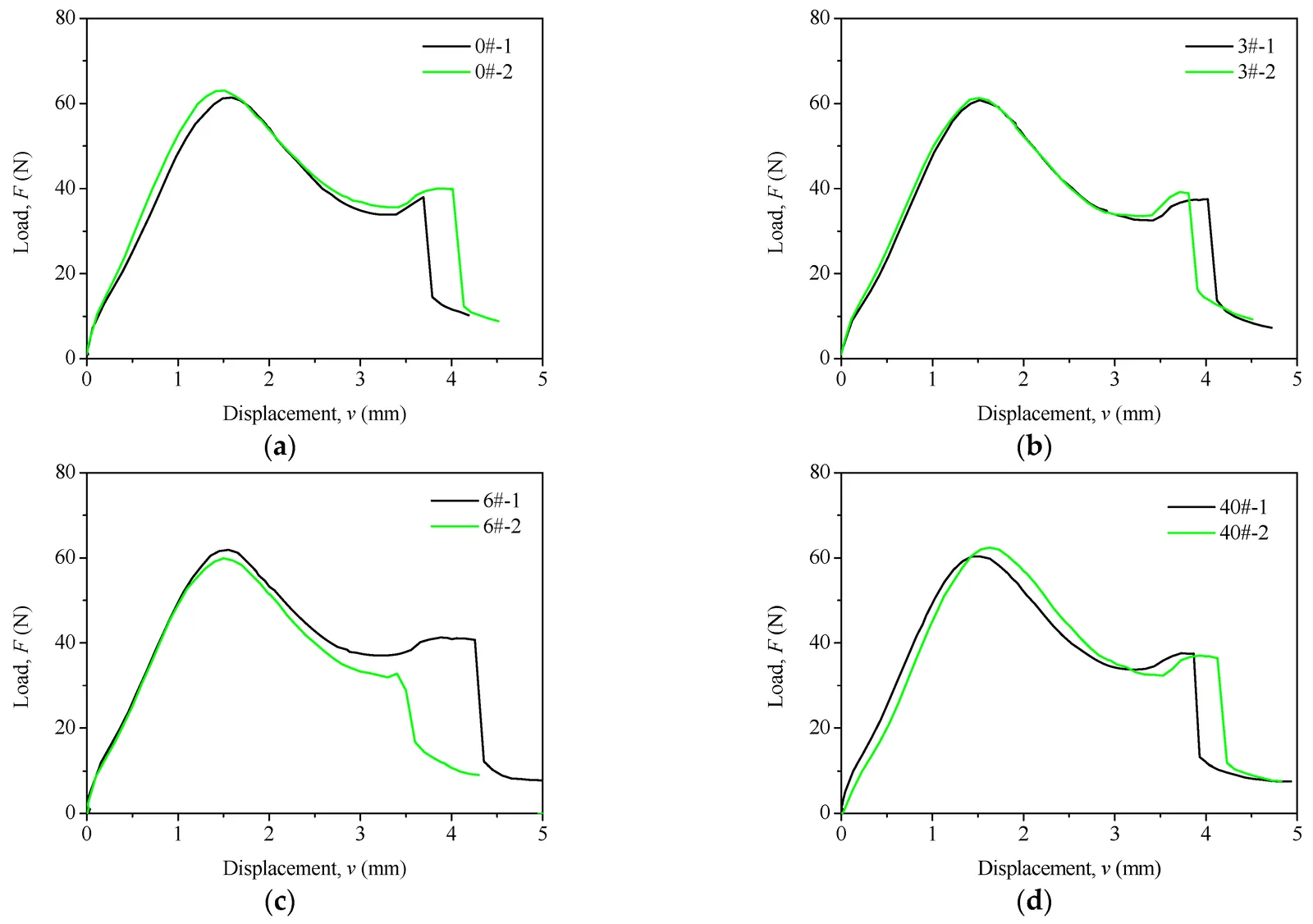
SPT results of load–displacement curves of HDPE pipe butt fusion welded joints at 29 °C in ambient air: (**a**) specimen 0#, (**b**) specimen 3#, (**c**) specimen 6#, and (**d**) specimen 40#.

The SPT results of load–displacement curves are shown in Figure 10 for all specimens. Every curve featured a single prominent peak load, followed by load reduction to a local minimum, and then a slight secondary load rise before final specimen ruptured. The overall curve shape matched the trends observed in simulations (Figure 5), with consistent magnitudes of $F_{m}$ and $v_{m}$. The values of $F_{m}$ and $v_{m}$ are listed in Table 4 for all specimens.

The average experimental yield stress of base HDPE measured via uniaxial tensile testing at 28 °C was 22.20 MPa [25]. SPT tests were conducted at 29 °C, only 1 °C higher, so the reference base-material yield stress at test temperature was approximated as 22.20 MPa. For specimen 40# (unaffected base material), calculated $\sigma_{y}$ decreased from 24.68 MPa (μ = 0.3) to 23.24 MPa (μ = 0.5), representing a 6% relative reduction. The value computed using μ = 0.5 (23.24 MPa) deviated by merely 5% from the tensile benchmark of 22.20 MPa, indicating that μ = 0.5 was the physically realistic friction coefficient for the present test fixture and HDPE samples. Therefore, all subsequent yield stress analyses adopt results calculated using Equation (4) (μ = 0.5).

Under μ = 0.5, as listed in Table 4, $\sigma_{y}$ in the weld center was 23.95 MPa, while the maximum value of 24.10 MPa occurred at 3 mm away from the weld center (3#). The base material (40#) exhibited the minimum yield stress of 23.24 MPa, representing a 4% strength reduction relative to the peak joint strength. This measurement explains why standard full-joint tensile specimens rarely fracture within the weld zone in prior literature [5,6,7,8]: properly fused joints possess higher yield resistance than the base HDPE matrix. In addition, all welding operations in this study strictly in accordance with ISO 21307 [26], thereby providing experimental evidence that adherence to standardized butt fusion procedures ensures the mechanical integrity of HDPE pipe butt fusion welded joints.

## 5. Conclusions

In this study, SPT combined with finite element parametric simulation to quantify the spatial distribution of yield stress across HDPE pipe butt fusion welded joints. The main conclusions of the study are as follows:

(1) Simulation results indicated that there was a strong linear correlation between HDPE yield stress and the ratio of maximum force to displacement at the point of maximum force. The contact friction coefficient between specimen and fixture imposed only minor influence on the empirical coefficients of the yield stress correlation formula, while material properties including yield stress and strain corresponding to the yield stress dominated the values of empirical coefficients. Comparative verification against uniaxial tensile test data indicated that the correlation formula calibrated under a friction coefficient μ = 0.5 delivered yield stress predictions with acceptable deviation for HDPE.
(2) Local SPT measurements revealed that the yield stress of HDPE butt fusion welded joints fabricated in accordance with ISO 21307 was universally higher than that of the base pipe material. The maximum yield stress emerged at a position 3 mm away from the weld center plane, with a 4% increment relative to the unaffected base HDPE matrix.
(3) SPT provides a feasible technical approach to evaluate the quality of butt fusion welded joints in polyethylene pipes. As SPT specimens require only a tiny amount of material for fabrication, the preparation process induces negligible damage to in-service pipelines. Accordingly, SPT is a reliable technique for quality assessment of butt fusion welded joints in operational polyethylene pipelines. Moreover, SPT exhibits considerable potential in characterizing the creep behavior of these welded joints, which is a critical mechanical property for predicting the long-term service performance of polyethylene pipelines. Future work will focus on adopting the SPT to investigate the creep performance of polyethylene pipe butt fusion welded joints.

## Figures and Tables

**Table 1:** Material constitutive parameters for all simulation cases.

Case	$\sigma_{y}$ (MPa)	$\varepsilon_{y}$ (%)
1	13.32	6.52
2	17.76	6.52
3	22.20	6.52
4	26.64	6.52
5	31.08	6.52
6	22.20	3.91
7	22.20	5.22
8	22.20	7.82
9	22.20	9.13

**Table 2:** $F_{m}$ and $v_{m}$ values for all simulation cases.

Case	$F_{m}$ (N)			$v_{m}$ (mm)		
	μ = 0.3	μ = 0.4	μ = 0.5	μ = 0.3	μ = 0.4	μ = 0.5
1	33.49	34.35	34.72	1.57	1.56	1.58
2	44.72	45.87	46.35	1.57	1.59	1.53
3	55.97	57.49	58.15	1.59	1.58	1.56
4	67.19	69.14	69.89	1.62	1.60	1.58
5	78.45	80.87	81.82	1.63	1.63	1.57
6	55.60	56.99	57.54	1.57	1.57	1.58
7	55.71	57.19	57.80	1.61	1.55	1.54
8	56.11	57.68	58.36	1.57	1.57	1.58
9	56.27	57.88	58.67	1.60	1.58	1.59

**Table 3:** Butt fusion welding parameters for HDPE pipes [27].

Heating Temperature (°C)	Endothermic Times (s)	Cooling Time (s)	Flanging Pressure (MPa)	Welding Pressure (MPa)
210	140	960	0.15	0.15

**Table 4:** SPT results of HDPE pipe butt fusion welded joints in ambient air at 29 °C.

Specimen	$F_{m}$ (N)	$v_{m}$ (mm)	Average $F_{m}$ (N)	Average $v_{m}$ (mm)	$\sigma_{y}$ (MPa)		
					μ = 0.3	μ = 0.4	μ = 0.5
0#-1	63.01	1.515	62.20	1.553	25.47	24.68	23.95
0#-2	61.41	1.591					
3#-1	61.32	1.508	61.05	1.514	25.64	24.83	24.10
3#-2	60.83	1.519					
6#-1	59.91	1.501	60.90	1.529	25.31	24.52	23.81
6#-2	61.92	1.556					
40#-1	62.41	1.626	61.35	1.578	24.68	23.92	23.24
40#-2	60.33	1.530					

## Data Availability

The original contributions presented in this study are included in the article. Further inquiries can be directed to the corresponding author.

## Nomenclature

*E*	Elastic modulus
*F*	Load
$F_{m}$	Maximum load
$h_{0}$	Initial thickness of the specimen
$\sigma_{y}$	Yield stress
$\varepsilon_{y}$	Strain at $\sigma_{y}$
μ	Friction coefficient
v	Punch displacement
$v_{m}$	v at $F_{m}$
$\beta_{0}$, $\beta_{\mathrm{Rm}}$	Empirical factors
